# Supplementary material for: Phenotypic characteristics of peripheral immune cells of Myalgic encephalomyelitis/chronic fatigue syndrome via transmission electron microscopy: A pilot study
Source: PLoS One. 2022 Aug 9;17(8):e0272703. doi: 10.1371/journal.pone.0272703 (PMC9362953; doi:10.1371/journal.pone.0272703)
Supplement: S1 Table — Isolated T cells were stimulated with anti-CD3/CD28 beads for 12 h and number of apoptotic and necrotic cells were measured based on morphological changes consistent with apoptotic or necrotic cell death by TEM at 200x or 500-1500X magnification. (DOCX) [file pone.0272703.s001.docx]

**Table S1.** **Quantitative analysis of transmission electron microscopy data on cellular apoptosis and necrosis in stimulated T cells.** Isolated T cells were stimulated with anti-CD3/CD28 beads for 12 h and number of apoptotic and necrotic cells were measured based on morphological changes consistent with apoptotic or necrotic cell death by TEM at 200x or 500-1500X magnification.

| **200x** | | | |
| --- | --- | --- | --- |
| Sample ID | Cells | Apoptotic | Necrotic |
|  |  |  |  |
| TCFS-T+Act | 1120 | 53 | 60 |
| THC-T+Act | 1289 | 43 | 64 |
| UCFS-T+Act | 1172 | 60 | 53 |
| UHC-T+Act | 1408 | 21 | 26 |
|  |  |  |  |
| **500-1500x** | | | |
| Sample ID | Cells | Apoptotic | Necrotic |
|  |  |  |  |
| TCFS-T+Act | 129 | 10 | 26 |
| THC-T+Act | 186 | 8 | 16 |
| UCFS-T+Act | 155 | 17 | 21 |
| UHC-T+Act | 130 | 3 | 7 |
