# Supplementary material for: Phenotypic characteristics of peripheral immune cells of Myalgic encephalomyelitis/chronic fatigue syndrome via transmission electron microscopy: A pilot study
Source: PLoS One. 2022 Aug 9;17(8):e0272703. doi: 10.1371/journal.pone.0272703 (PMC9362953; doi:10.1371/journal.pone.0272703)
Supplement: S2 Table — Fisher’s exact test of the 2x2 contingency table was used to assess the significance of the proportion differences between apoptosis and necrosis in stimulated T cells between ME/CFS and healthy control at 500-1500X magnification. (DOCX) [file pone.0272703.s002.docx]

| **Table S2.** **Statistical analyses of transmission electron microscopy data on T cell death following immune activation.** Fisher's exact test of the 2x2 contingency table was used to assess the significance of the proportional differences between apoptosis and necrosis in stimulated T cells between ME/CFS and healthy control at 500-1500X magnification.   \| **500-1500x** \| \| \| \| \| \| --- \| --- \| --- \| --- \| --- \| \| **Contingency table** \|  \|  \|  \|  \| \|  \| Apoptotic \| Necrotic \| Healthy live cells \|  \| \|  \|  \|  \|  \|  \| \| ME/CFS \| 27 \| 47 \| 210 \|  \| \| HC \| 11 \| 23 \| 282 \|  \| \|  \|  \|  \|  \|  \| \|  \|  \|  \|  \|  \| \| **Fisher’s Exact Test** \|  \|  \|  \|  \| \|  \|  \|  \|  \|  \| \| Apoptotic \| Odd’s Ratio \| 3.288821 \|  \|  \| \|  \| P-Value \| 0.001045 \|  \|  \| \|  \|  \|  \|  \|  \| \| Necrotic \| Odd’s Ratio \| 2.739168 \|  \|  \| \|  \| P-Value \| 0.0001679 \|  \|  \| \|  \|  \|  \|  \|  \| |
| --- | --- | --- | --- | --- | --- | --- | --- | --- | --- | --- | --- | --- | --- | --- | --- | --- | --- | --- | --- | --- | --- | --- | --- | --- | --- | --- | --- | --- | --- | --- | --- | --- | --- | --- | --- | --- | --- | --- | --- | --- | --- | --- | --- | --- | --- | --- | --- | --- | --- | --- | --- | --- | --- | --- | --- | --- | --- | --- | --- | --- | --- | --- | --- | --- | --- | --- | --- | --- | --- | --- | --- | --- | --- | --- | --- | --- | --- | --- | --- | --- |
