# Supplementary material for: Phenotypic characteristics of peripheral immune cells of Myalgic encephalomyelitis/chronic fatigue syndrome via transmission electron microscopy: A pilot study
Source: PLoS One. 2022 Aug 9;17(8):e0272703. doi: 10.1371/journal.pone.0272703 (PMC9362953; doi:10.1371/journal.pone.0272703)
Supplement: S3 Table — Fisher’s exact test of the 2x2 contingency table was used to assess the significance of the proportion differences between apoptosis and necrosis in stimulated T cells between the identical twins discordant with moderate form of ME/CFS or unrelated participants discordant with extreme form of ME/CFS. This was measured by TEM at 200x or 500-1500X magnification. (DOCX) [file pone.0272703.s003.docx]

**Table S3.** **Statistical analyses of transmission electron microscopy data on T cell death following immune activation within each pair (identical twin or unrelated pair).** Fisher's exact test of the 2x2 contingency table was used to assess the significance of the proportion differences between apoptosis and necrosis in stimulated T cells between the identical twins discordant with moderate form of ME/CFS or unrelated participants discordant with extreme form of ME/CFS. This was measured by TEM at 200x or 500-1500X magnification.

| **200x** | | | | |
| --- | --- | --- | --- | --- |
| **Contingency table** |  |  |  |  |
| Sample ID | Apoptotic | Necrotic | Healthy live cells |  |
|  |  |  |  |  |
| UCFS | 60 | 53 | 1059 |  |
| UHC | 21 | 26 | 1361 |  |
|  |  |  |  |  |
| TCFS | 53 | 60 | 1007 |  |
| THC | 43 | 64 | 1059 |  |
|  |  |  |  |  |
| **Fisher’s Exact Test** |  |  |  |  |
|  |  |  |  |  |
| **Unrelated** | Apoptotic | Odd’s Ratio | 3.670102 |  |
|  |  | P-Value | 7.76e-08 |  |
|  |  |  |  |  |
|  | Necrotic | Odd’s Ratio | 2.618791 |  |
|  |  | P-Value | 4.614e-05 |  |
|  |  |  |  |  |
| **Twin** | Apoptotic | Odd’s Ratio | 1.296045 |  |
|  |  | P-Value | 0.2506 |  |
|  |  |  |  |  |
|  | Necrotic | Odd’s Ratio | 1.190437 |  |
|  |  | P-Value | 1 |  |
|  |  |  |  |  |

| **500-1500x** | | | | |
| --- | --- | --- | --- | --- |
| **Contingency table** |  |  |  |  |
| Sample ID | Apoptotic | Necrotic | Normal cells |  |
|  |  |  |  |  |
| UCFS | 17 | 21 | 117 |  |
| UHC | 3 | 7 | 120 |  |
|  |  |  |  |  |
| TCFS | 10 | 26 | 93 |  |
| THC | 8 | 16 | 162 |  |
|  |  |  |  |  |
| **Fisher’s Exact Test** |  |  |  |  |
|  |  |  |  |  |
| **Unrelated** | Apoptotic | Odd’s Ratio | 5.778141 |  |
|  |  | P-Value | 0.002094 |  |
|  |  |  |  |  |
|  | Necrotic | Odd’s Ratio | 3.064727 |  |
|  |  | P-Value | 0.01517 |  |
|  |  |  |  |  |
| **Twin** | Apoptotic | Odd’s Ratio | 2.170918 |  |
|  |  | P-Value | 0.1321 |  |
|  |  |  |  |  |
|  | Necrotic | Odd’s Ratio | 2.820232 |  |
|  |  | P-Value | 0.002227 |  |
