# Supplementary material for: Phenotypic characteristics of peripheral immune cells of Myalgic encephalomyelitis/chronic fatigue syndrome via transmission electron microscopy: A pilot study
Source: PLoS One. 2022 Aug 9;17(8):e0272703. doi: 10.1371/journal.pone.0272703 (PMC9362953; doi:10.1371/journal.pone.0272703)
Supplement: S4 Table — Fisher’s exact test of the 2x2 contingency table to assess the significance of the proportion differences between apoptosis and necrosis in PBMC subpopulation lacking T cells. Cells were incubated in the presence or absence of 100nM PMA for 12 h and number of apoptotic and necrotic cells were measured in unstimulated and PMA-stimulated cells by TEM at 200-1500X magnification, based on morphological changes consistent with apoptotic or necrotic cell death. (DOCX) [file pone.0272703.s004.docx]

**Table S5. Quantitative analysis of transmission electron microscopy data on mitochondrial ultrastructural abnormalities in stimulated T cells from ME/CFS patients or healthy controls.** Isolated T cells were stimulated with anti-CD3/CD28 beads for 12 h. Mitochondria were counted per cell and assessed for morphological changes (normal, vesicular/compartmentalized or swollen). MT with vesicular/ compartmentalized and swollen morphologies were considered abnormal. This was measured by TEM at 300-2500x magnification.

|  | **Total count** | | | | | |
| --- | --- | --- | --- | --- | --- | --- |
| Sample ID | Cell | MT | Normal MT | Vesicular/  compartmentalized | Swollen | Abnormal MT (vesicular/ compartmentalized + swollen) |
| TCSF-T+Act | 22 | 173 | 93 | 42 | 38 | 80 |
| THC-T+Act | 56 | 487 | 324 | 110 | 53 | 163 |
| UCFS-T+Act | 18 | 191 | 116 | 50 | 25 | 75 |
| UHC-T+Act | 38 | 282 | 187 | 78 | 17 | 95 |
|  |  |  |  |  |  |  |
|  | **Average±STDEV** | | | | | |
| Sample ID |  | MT count per cell | Normal MT | Vesicular/  compartmentalized | Swollen | Abnormal MT (vesicular/ compartmentalized + swollen) |
| TCSF-T+Act |  | 7.7±2.40 | 4.2±1.72 | 1.9±1.31 | 1.7±1.35 | 3.6±2.15 |
| THC-T+Act |  | 8.7±4.57 | 5. 8±3.34 | 2.0±1.74 | 0.9±1.07 | 2.9±2.03 |
| UCFS-T+Act |  | 10.6±3.92 | 6.4±2.73 | 2.8±1.86 | 1.4±1.60 | 4.2±3.08 |
| UHC-T+Act |  | 7.8±3.613 | 5.2±2.70 | 2.2±1.44 | 0.5±0.70 | 2.6±1.82 |
|  |  |  |  |  |  |  |
