# Supplementary material for: Phenotypic characteristics of peripheral immune cells of Myalgic encephalomyelitis/chronic fatigue syndrome via transmission electron microscopy: A pilot study
Source: PLoS One. 2022 Aug 9;17(8):e0272703. doi: 10.1371/journal.pone.0272703 (PMC9362953; doi:10.1371/journal.pone.0272703)
Supplement: S5 Table — Isolated T cells were stimulated with anti-CD3/CD28 beads for 12 h. Mitochondria were counted per cell and assessed for morphological changes (normal, vesicular/compartmentalized or swollen). MT with vesicular/ compartmentalized and swollen morphologies were considered abnormal. This was measured by TEM at 300-2500x magnification. (DOCX) [file pone.0272703.s005.docx]

**Table S4. Statistical analyses of transmission electron microscopy data on cell death in unstimulated and stimulated isolated PBMC subpopulation lacking T cells.** Fisher's exact test of the 2x2 contingency table to assess the significance of the proportion differences between apoptosis and necrosis in PBMC subpopulation lacking T cells. Cells were incubated in the presence or absence of 100nM PMA for 12 h and number of apoptotic and necrotic cells were measured in unstimulated and PMA-stimulated cells by TEM at 200-1500X magnification, based on morphological changes consistent with apoptotic or necrotic cell death.

| **Unstimulated PBMC subpopulation lacking T cells** | | | |
| --- | --- | --- | --- |
| Sample ID | Cells | Apoptotic | Necrotic |
|  |  |  |  |
| TCFS-P-T | 127 | 5 | 22 |
| THC-P-T | 91 | 10 | 7 |
| UHC-P-T | 128 | 8 | 16 |
|  |  |  |  |
|  |  |  |  |
| **Stimulated PBMC subpopulation lacking T cells** | | | |
| Sample ID | Cells | Apoptotic | Necrotic |
|  |  |  |  |
| UCFS-P-T+Act | 587 | 42 | 60 |
| UHC-P-T+Act | 642 | 59 | 45 |
|  |  |  |  |
|  |  |  |  |

| **Unstimulated PBMC subpopulation lacking T cells** | | | | |
| --- | --- | --- | --- | --- |
| **Contingency table** |  |  |  |  |
| Sample ID | Apoptotic | Necrotic | Normal |  |
|  |  |  |  |  |
| TCFS-P-T | 5 | 22 | 100 |  |
| THC-P-T | 10 | 7 | 74 |  |
| UHC-P-T | 8 | 11 | 104 |  |
|  |  |  |  |  |
|  |  |  |  |  |
| **Fisher’s Exact Test** |  |  |  |  |
|  |  |  |  |  |
| **Twin ME/CFS vs Twin HC** | Apoptotic | Odd’s Ratio | 0.3719502 |  |
|  |  | P-Value | 0.1028 |  |
|  |  |  |  |  |
|  | Necrotic | Odd’s Ratio | 2.316691 |  |
|  |  | P-Value | 0.06781 |  |
|  |  |  |  |  |
| **Twin ME/CFS vs Unrelated HC** | Apoptotic | Odd’s Ratio | 0.6512744 |  |
|  |  | P-Value | 0.5718 |  |
|  |  |  |  |  |
|  | Necrotic | Odd’s Ratio | 2.073732 |  |
|  |  | P-Value | 0.06361 |  |
|  |  |  |  |  |

| **Stimulated PBMC subpopulation lacking T cells** | | | | |
| --- | --- | --- | --- | --- |
| **Contingency table** |  |  |  |  |
| Sample ID | Apoptotic | Necrotic | Healthy live cells |  |
| UCFS-P-T+act | 42 | 60 | 485 |  |
| UHC-P-T+act | 59 | 45 | 538 |  |
|  |  |  |  |  |
|  |  |  |  |  |
| **Fisher’s Exact Test** |  |  |  |  |
|  |  |  |  |  |
| **Unrelated** | Apoptotic | Odd’s Ratio | 0.789822 |  |
|  |  | P-Value | 0.2963 |  |
|  |  |  |  |  |
|  | Necrotic | Odd’s Ratio | 1.47851 |  |
|  |  | P-Value | 0.06483 |  |
|  |  |  |  |  |
