# Supplementary material for: Phenotypic characteristics of peripheral immune cells of Myalgic encephalomyelitis/chronic fatigue syndrome via transmission electron microscopy: A pilot study
Source: PLoS One. 2022 Aug 9;17(8):e0272703. doi: 10.1371/journal.pone.0272703 (PMC9362953; doi:10.1371/journal.pone.0272703)
Supplement: S6 Table — Fisher’s exact test of the 2x2 contingency table was used to assess the significance of the proportion differences between variations in mitochondrial morphology (normal, vesicular/compartmentalized or swollen), with abnormal mitochondria being the sum of vesicular/compartmentalized and swollen per sample. This was performed in stimulated T cells isolated from an identical twin discordant with moderate ME/CFS and a pair of unrelated participants discordant with extreme form of ME/CFS. (DOCX) [file pone.0272703.s006.docx]

**Table S6. Statistical analyses of transmission electron microscopy data on mitochondrial ultrastructural abnormalities in stimulated T cells.** Fisher's exact test of the 2x2 contingency table was used to assess the significance of the proportion differences between variations in mitochondrial morphology (normal, vesicular/compartmentalized or swollen), with abnormal mitochondria being the sum of vesicular/compartmentalized and swollen per sample. This was performed in stimulated T cells isolated from an identical twin discordant with moderate ME/CFS and a pair of unrelated participants discordant with extreme form of ME/CFS.

|  | | | | |
| --- | --- | --- | --- | --- |
| **Contingency table** |  |  |  |  |
| Sample ID | Vesicular/  Compartmentalized | Swollen | Abnormal | Normal |
|  |  |  |  |  |
| ME/CFS | 92 | 63 | 155 | 209 |
| HC | 188 | 70 | 285 | 511 |
|  |  |  |  |  |
| TCFS | 42 | 38 | 80 | 93 |
| THC | 110 | 53 | 163 | 324 |
|  |  |  |  |  |
| UCFS | 50 | 25 | 75 | 116 |
| UHC | 78 | 17 | 95 | 187 |
|  |  |  |  |  |
| **Fisher’s Exact Test** |  |  |  |  |
|  |  |  |  |  |
| **Total ME/CFS and Healthy controls** | Vesicular/  Compartmentalized | Odd’s Ratio | 1.196243 |  |
|  |  | P-Value | 0.2497 |  |
|  |  |  |  |  |
|  | Swollen | Odd’s Ratio | 2.198272 |  |
|  |  | P-Value | 4.77e-05 |  |
|  |  |  |  |  |
|  | Abnormal | Odd’s Ratio | 1.468315 |  |
|  |  | P-Value | 0.003622 |  |
|  |  |  |  |  |
| **Twins** | Vesicular/  Compartmentalized | Odd’s Ratio | 1.329552 |  |
|  |  | P-Value | 0.2203 |  |
|  |  |  |  |  |
|  | Swollen | Odd’s Ratio | 2.492796 |  |
|  |  | P-Value | 0.0003007 |  |
|  |  |  |  |  |
|  | Abnormal | Odd’s Ratio | 1.708489 |  |
|  |  | P-Value | 0.003297 |  |
|  |  |  |  |  |
| **Unrelated** | Vesicular/  Compartmentalized | Odd’s Ratio | 1.03332 |  |
|  |  | P-Value | 0.9139 |  |
|  |  |  |  |  |
|  | Swollen | Odd’s Ratio | 2.36455 |  |
|  |  | P-Value | 0.01153 |  |
|  |  |  |  |  |
|  | Abnormal | Odd’s Ratio | 1.272015 |  |
|  |  | P-Value | 0.2414 |  |
