# Supplementary material for: Phenotypic characteristics of peripheral immune cells of Myalgic encephalomyelitis/chronic fatigue syndrome via transmission electron microscopy: A pilot study
Source: PLoS One. 2022 Aug 9;17(8):e0272703. doi: 10.1371/journal.pone.0272703 (PMC9362953; doi:10.1371/journal.pone.0272703)
Supplement: S7 Table — The severity in mitochondrial dysfunction was assessed via calculating the percentage of cells per each group carrying more than 3 swollen or 6 abnormal mitochondria per single cell. (DOCX) [file pone.0272703.s007.docx]

**Table S7. Quantitative analysis of transmission electron microscopy data on mitochondrial ultrastructural abnormalities in activated T cells.** The severity in mitochondrial dysfunction was assessed via calculating the percentage of cells per each group carrying more than 3 swollen or 6 abnormal mitochondria per single cell.

|  | | **Total count** | | | | **Ratio** | | | | | **%** | | |
| --- | --- | --- | --- | --- | --- | --- | --- | --- | --- | --- | --- | --- | --- |
| Sample ID | | Cell | | Cells with ≥3 Swollen MT per cells | Cells with ≥6 Abnormal MT per cell (vesicular/ compartmentalized + swollen) | | | Cells with ≥3 Swollen MT per cells | Cells with≥6 abnormal MT (vesicular/ compartmentalized + swollen) | Cells with≥3 swollen MT | | | Cells with≥6 abnormal MT (vesicular/ compartmentalized + swollen) |
| TCSF-T+Act | | 22 | | 5 | 4 | | | 0.23 | 0.18 | 22.7 | | | 18.2 |
| THC-T+Act | | 56 | | 5 | 5 | | | 0.089 | 0.09 | 8.9 | | | 8.9 |
| UCFS-T+Act | | 18 | | 5 | 7 | | | 0.28 | 0.39 | 27.8 | | | 38.9 |
| UHC-T+Act | | 38 | | 0 | 3 | | | 0 | 0.08 | 0 | | | 7.9 |
|  |  |  |  | |  | |  | | | |  |  | |
