# Supplementary material for: Phenotypic characteristics of peripheral immune cells of Myalgic encephalomyelitis/chronic fatigue syndrome via transmission electron microscopy: A pilot study
Source: PLoS One. 2022 Aug 9;17(8):e0272703. doi: 10.1371/journal.pone.0272703 (PMC9362953; doi:10.1371/journal.pone.0272703)
Supplement: S8 Table — Fisher’s exact test of the 2x2 contingency table was used to assess the significance of the proportion difference in severity in mitochondrial dysfunction based on the presence of ≥ 3 swollen or 6 abnormal mitochondria per single cell. (DOCX) [file pone.0272703.s008.docx]

**Table S8. Statistical analyses of transmission electron microscopy data on the severity of mitochondrial ultrastructural abnormalities in stimulated T cells.** Fisher's exact test of the 2x2 contingency table was used to assess the significance of the proportion difference in severity in mitochondrial dysfunction based on the presence of ≥ 3 swollen or 6 abnormal mitochondria per single cell.

| **≥ 3 swollen MT per cells between ME/CFS and HC** | | | | |
| --- | --- | --- | --- | --- |
| **Contingency table** |  |  |  |  |
|  | ≥ 3 swollen MT per cells | ≤ 3 swollen MT per cells |  |  |
|  |  |  |  |  |
| ME/CFS | 10 | 30 |  |  |
| HC | 5 | 89 |  |  |
|  |  |  |  |  |
| **Fisher’s Exact Test** |  |  |  |  |
|  |  |  |  |  |
|  | Odd’s Ratio | 5.838787 |  |  |
|  | P-Value | 0.001989 |  |  |
|  |  |  |  |  |

| **≥ 6 abnormal MT per cells between ME/CFS and HC** | | | | |
| --- | --- | --- | --- | --- |
| **Contingency table** |  |  |  |  |
|  | ≥ 6 swollen MT per cells | ≤ 6 swollen MT per cells |  |  |
|  |  |  |  |  |
| ME/CFS | 11 | 29 |  |  |
| HC | 8 | 86 |  |  |
|  |  |  |  |  |
|  |  |  |  |  |
| **Fisher’s Exact Test** |  |  |  |  |
|  |  |  |  |  |
|  | Odd’s Ratio | 4.026865 |  |  |
|  | P-Value | 0.006459 |  |  |
|  |  |  |  |  |

| **≥ 3 swollen MT per cells between UCFS and UHC** | | | | |
| --- | --- | --- | --- | --- |
| **Contingency table** |  |  |  |  |
|  | ≥ 3 swollen MT per cells | ≤ 3 swollen MT per cells |  |  |
|  |  |  |  |  |
| UCFS | 5 | 13 |  |  |
| UHC | 0 | 38 |  |  |
|  |  |  |  |  |
| **Fisher’s Exact Test** |  |  |  |  |
|  |  |  |  |  |
|  | Odd’s Ratio | Inf |  |  |
|  | P-Value | 0.002243 |  |  |
|  |  |  |  |  |

| **≥ 6 Abnormal MT per cells between UCFS and UHC** | | | | |
| --- | --- | --- | --- | --- |
| **Contingency table** |  |  |  |  |
|  | ≥ 6 abnormal MT per cells | ≤ 6 abnormal MT per cells |  |  |
|  |  |  |  |  |
| UCFS | 7 | 11 |  |  |
| UHC | 3 | 35 |  |  |
|  |  |  |  |  |
| **Fisher’s Exact Test** |  |  |  |  |
|  |  |  |  |  |
|  | Odd’s Ratio | 7.105461 |  |  |
|  | P-Value | 0.008457 |  |  |
|  |  |  |  |  |
