# Supplementary material for: Phenotypic characteristics of peripheral immune cells of Myalgic encephalomyelitis/chronic fatigue syndrome via transmission electron microscopy: A pilot study
Source: PLoS One. 2022 Aug 9;17(8):e0272703. doi: 10.1371/journal.pone.0272703 (PMC9362953; doi:10.1371/journal.pone.0272703)
Supplement: S9 Table — Intracellular and extracellular lipid droplets-like vesicles were counted in unstimulated and stimulated PBMC subpopulation from TEM micrographs at 300 to 800x. (DOCX) [file pone.0272703.s009.docx]

**Table S9.** **Quantitative analysis of transmission electron microscopy data on intracellular and extracellular lipid droplets-like vesicles.** Intracellular and extracellular lipid droplets-like vesicles were counted in unstimulated and stimulated PBMC subpopulation from TEM micrographs at 300 to 800x.

| **300 – 800x** | | | | |
| --- | --- | --- | --- | --- |
| Sample ID | Cell count | Intracellular lipid droplet-like vesicles | Extracellular lipid droplet-like vesicles |  |
|  |  |  |  |  |
| TCFS-T+act | 84 | 0 | 0 |  |
| THC-T+act | 152 | 0 | 0 |  |
| UCFS-T+act | 140 | 3 | 0 |  |
| UHC-T+act | 83 | 0 | 0 |  |
|  |  |  |  |  |
| TCFS-P-T | 108 | 0 | 0 |  |
| THC-P-T | 70 | 1 | 0 |  |
| UHC-P-T | 114 | 1 | 0 |  |
|  |  |  |  |  |
| UCFS-P-T+act | 460 | 36 | 59 |  |
| UHC-P-T+act | 530 | 9 | 40 |  |
|  |  |  |  |  |
