# Supplementary material for: Phenotypic characteristics of peripheral immune cells of Myalgic encephalomyelitis/chronic fatigue syndrome via transmission electron microscopy: A pilot study
Source: PLoS One. 2022 Aug 9;17(8):e0272703. doi: 10.1371/journal.pone.0272703 (PMC9362953; doi:10.1371/journal.pone.0272703)
Supplement: S10 Table — Fisher’s exact test of the 2x2 contingency table was used to assess the significance of the proportion differences in intracellular and extracellular lipid droplet-like vesicles in stimulated PBMC subpopulation lacking T cells between unrelated extremely severe ME/CFS patient and unrelated healthy control. (DOCX) [file pone.0272703.s010.docx]

**Table S10. Statistical analyses of transmission electron microscopy data on intracellular and extracellular lipid droplets-like vesicles.** Fisher's exact test of the 2x2 contingency table was used to assess the significance of the proportion differences in intracellular and extracellular lipid droplet-like vesicles in stimulated PBMC subpopulation lacking T cells between unrelated extremely severe ME/CFS patient and unrelated healthy control.

|  | | | | |
| --- | --- | --- | --- | --- |
| **Contingency table** |  |  |  |  |
| Sample ID | Intracellular lipid droplet-like vesicle | Extracellular lipid droplet-like vesicle |  |  |
|  |  |  |  |  |
| UCFS | 36 | 59 |  |  |
| UHC | 9 | 40 |  |  |
|  |  |  |  |  |
|  |  |  |  |  |
| **Fisher’s Exact Test** |  |  |  |  |
|  |  |  |  |  |
| Intracellular lipid droplet | Odd’s Ratio | 2.694116 |  |  |
|  | P-Value | 0.02225 |  |  |
