# Supplementary material for: Phenotypic characteristics of peripheral immune cells of Myalgic encephalomyelitis/chronic fatigue syndrome via transmission electron microscopy: A pilot study
Source: PLoS One. 2022 Aug 9;17(8):e0272703. doi: 10.1371/journal.pone.0272703 (PMC9362953; doi:10.1371/journal.pone.0272703)
Supplement: S11 Table — Giant platelet, platelet clump and giant rosette like-platelet aggregate were counted in stimulated and unstimulated PBMC subpopulation from TEM micrographs. (DOCX) [file pone.0272703.s011.docx]

**Table S11. Quantitative analysis of transmission electron microscopy data on giant platelet, platelet clump and giant rosette like-platelet aggregate.** Giant platelet, platelet clump and giant rosette like-platelet aggregate were counted in stimulated and unstimulated PBMC subpopulation from TEM micrographs.

| **Stimulated T Cells** | | | | |
| --- | --- | --- | --- | --- |
| Sample ID | Number of cells without platelets | Giant platelet | Platelet clump | Giant rosette-like platelet aggregate |
|  |  |  |  |  |
| TCFS-T+Act | 129 | 26 | 2 | 2 |
| THC-T+Act | 186 | 51 | 5 | 0 |
| UCFS-T+Act | 155 | 39 | 15 | 3 |
| UHC-T+Act | 130 | 15 | 6 | 2 |
|  |  |  |  |  |
|  |  |  |  |  |
| **PBMC subpopulation lacking T Cells** | | | | |
| Sample ID | Number of cells without platelets | Giant platelet | Platelet clump | Giant rosette-like platelet aggregate |
|  |  |  |  |  |
| TCFS-P-T | 127 | 40 | 16 | 0 |
| THC-P-T | 91 | 69 | 37 | 0 |
| UHC-P-T | 128 | 55 | 40 | 0 |
|  |  |  |  |  |
|  |  |  |  |  |
| **Stimulated PBMC subpopulation lacking T Cells** | | | | |
| Sample ID | Number of cells without platelets | Giant platelet | Platelet clump | Giant rosette-like platelet aggregate |
|  |  |  |  |  |
| UCFS-P-T+Act | 587 | 123 | 1 | 0 |
| UHC-P-T+Act | 642 | 131 | 4 | 0 |
|  |  |  |  |  |
