# Supplementary material for: Phenotypic characteristics of peripheral immune cells of Myalgic encephalomyelitis/chronic fatigue syndrome via transmission electron microscopy: A pilot study
Source: PLoS One. 2022 Aug 9;17(8):e0272703. doi: 10.1371/journal.pone.0272703 (PMC9362953; doi:10.1371/journal.pone.0272703)
Supplement: S12 Table — Fisher’s exact test of the 2x2 contingency table to assess the significance of the proportion differences between giant platelet, platelet clump and giant rosette-like platelet aggregate counts in unstimulated and stimulated PBMC subpopulation between ME/CFS and healthy controls. (DOCX) [file pone.0272703.s012.docx]

**Table S12. Statistical analyses of transmission electron microscopy data on giant platelet, platelet clump and giant rosette-like platelet aggregate.** Fisher's exact test of the 2x2 contingency table to assess the significance of the proportion differences between giant platelet, platelet clump and giant rosette-like platelet aggregate counts in unstimulated and stimulated PBMC subpopulation between ME/CFS and healthy controls.

| **Stimulated T cells** | | | | |
| --- | --- | --- | --- | --- |
| **Contingency table** |  |  |  |  |
|  | Giant platelet | Platelet clump | Giant rosette-like platelet aggregate | Non-platelets Cells |
|  |  |  |  |  |
| ME/CFS | 65 | 17 | 5 | 284 |
| HC | 66 | 11 | 2 | 316 |
|  |  |  |  |  |
|  |  |  |  |  |
| **Fisher’s Exact Test** |  |  |  |  |
|  |  |  |  |  |
| Giant platelet | Odd’s Ratio | 1.095679 |  |  |
|  | P-Value | 0.6995 |  |  |
|  |  |  |  |  |
| Platelet clump | Odd’s Ratio | 1.718099 |  |  |
|  | P-Value | 0.1801 |  |  |
|  |  |  |  |  |
| Giant rosette-like platelet aggregate | Odd’s Ratio | 2.777256 |  |  |
|  | P-Value | 0.2664 |  |  |
|  |  |  |  |  |
