# Supplementary material for: Phenotypic characteristics of peripheral immune cells of Myalgic encephalomyelitis/chronic fatigue syndrome via transmission electron microscopy: A pilot study
Source: PLoS One. 2022 Aug 9;17(8):e0272703. doi: 10.1371/journal.pone.0272703 (PMC9362953; doi:10.1371/journal.pone.0272703)
Supplement: S13 Table — Fisher’s exact test of the 2x2 contingency table to assess the significance of the proportion differences between giant platelet, platelet clump and giant rosette-like platelet aggregate counts in unstimulated and stimulated PBMC subpopulation between unrelated extremely severe ME/CFS and unrelated healthy control. (DOCX) [file pone.0272703.s013.docx]

**Table S13. Statistical analyses of transmission electron microscopy data on giant platelet, platelet clump and giant rosette-like platelet aggregate in the unrelated pair.** Fisher's exact test of the 2x2 contingency table to assess the significance of the proportion differences between giant platelet, platelet clump and giant rosette-like platelet aggregate counts in unstimulated and stimulated PBMC subpopulation between unrelated extremely severe ME/CFS and unrelated healthy control.

| **Stimulated PBMC subpopulation lacking T cells** | | | | |
| --- | --- | --- | --- | --- |
| **Contingency table** |  |  |  |  |
| Sample ID | Giant platelet | Platelet clump | Giant rosette-like platelet aggregate | Cells without platelets |
|  |  |  |  |  |
| UCFS | 39 | 15 |  | 155 |
| UHC | 15 | 6 |  | 130 |
|  |  |  |  |  |
|  |  |  |  |  |
| **Fisher’s Exact Test** |  |  |  |  |
|  |  |  |  |  |
| Giant platelet | Odd’s Ratio | 2.175891 |  |  |
|  | P-Value | 0.01643 |  |  |
|  |  |  |  |  |
| Platelet clump | Odd’s Ratio | 2.092009 |  |  |
|  | P-Value | 0.1725 |  |  |
