# Supplementary figures and images for: Phenotypic characteristics of peripheral immune cells of Myalgic encephalomyelitis/chronic fatigue syndrome via transmission electron microscopy: A pilot study
Source: PLoS One. 2022 Aug 9;17(8):e0272703. doi: 10.1371/journal.pone.0272703 (PMC9362953; doi:10.1371/journal.pone.0272703)

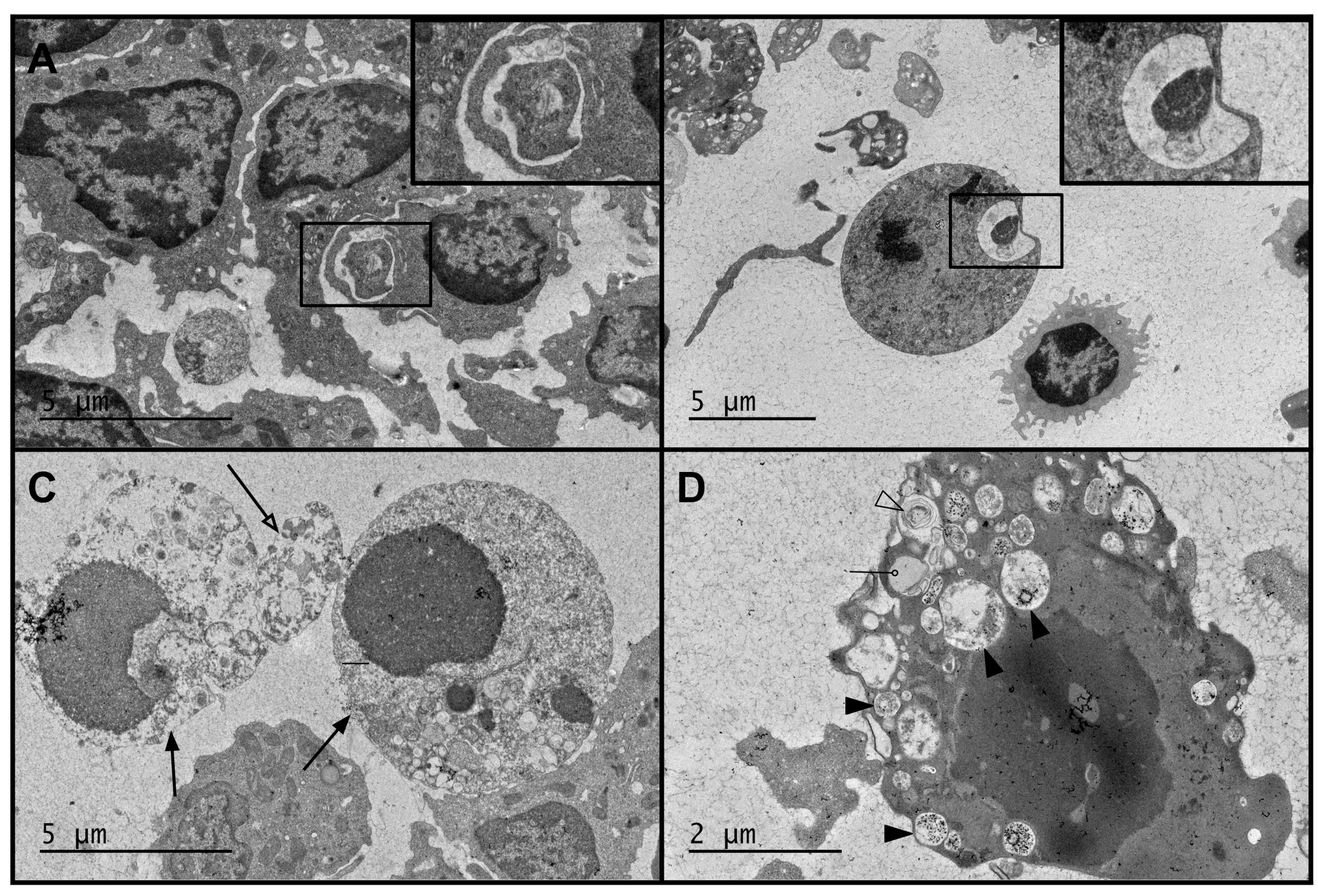

Supplement: S1 Fig — A) TEM image displaying immune synapse formation between a few PBMCs. Note the cell in the center has phagocytosed a platelet, (inset) shows how the plasma membrane of cell forms a pocket to engulf the platelet. B) A phagosome, which contains a large electron dense particle, 1 μm in diameter. C) Two apoptotic cells (arrow), with the apoptotic cell on the left displaying a large apoptotic body (open arrow). D) Cell undergoing autophagic cell death, demonstrating autophagic-like vacuoles, filled with the amorphous materials (arrowheads), the membranous inclusions (open arrowheads) or the organelles (circle) at the various stages of degradation. (TIF) [file pone.0272703.s014.tif]

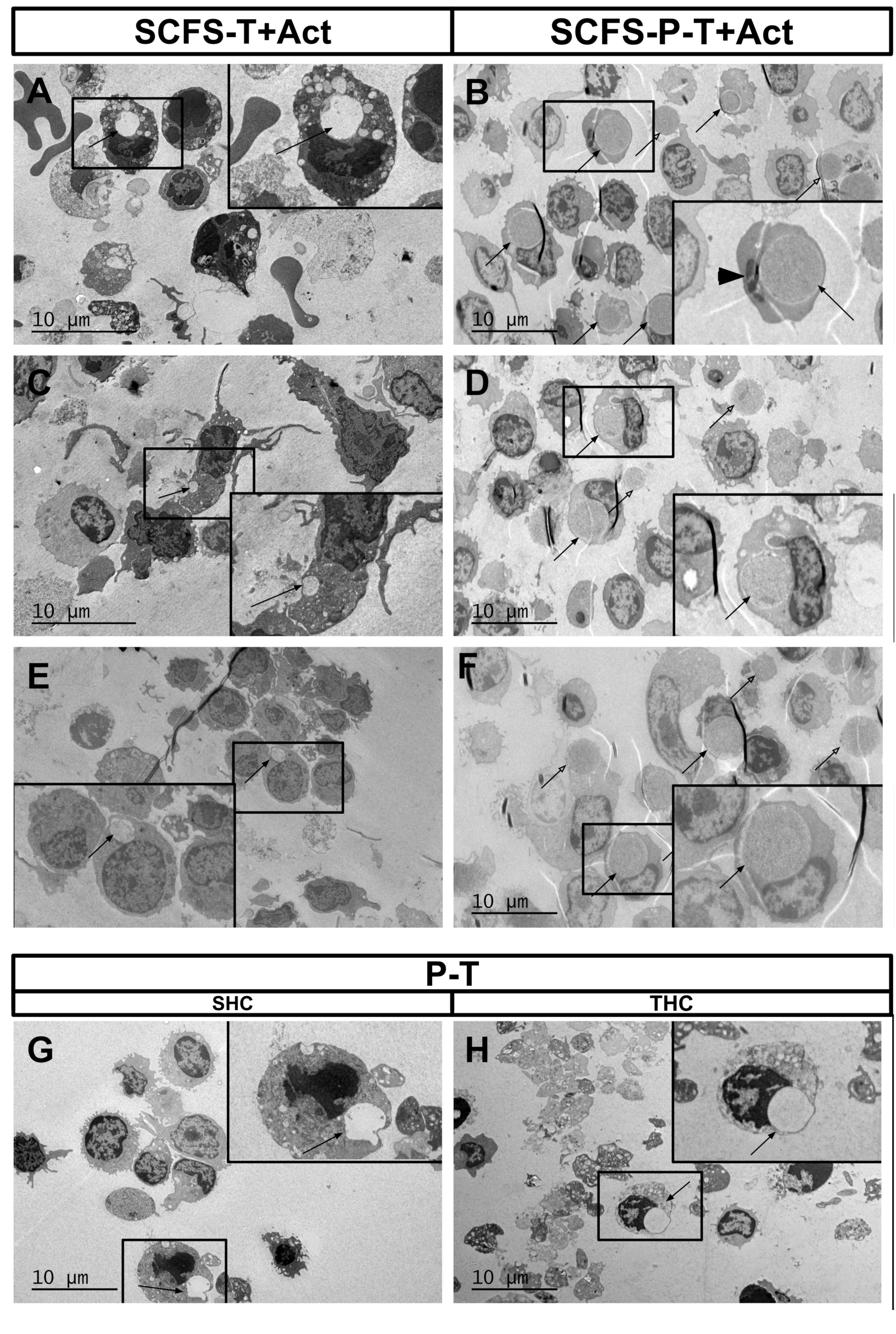

Supplement: S2 Fig — (A, C, E) Representative images of lipid droplet-like vesicles (arrow) in stimulated T cells from unrelated extremely severe ME/CFS patient (UCFS-T+Act). (B, D, F) Representative images of intracellular (arrow) and extracellular (open arrow) “giant lipid droplet-like vesicles” in stimulated PBMC lacking T cells from unrelated extremely severe ME/CFS (UCFS-P-T+Act). Note the difference in morphology and electron density of these “giant lipid droplet-like vesicles” in comparison to the unstimulated PBMC lacking T (P-T) cells and stimulated T cells (T+Act). (B) A giant lipid droplet-like vesicle compressing the nucleus of the cell (arrowhead). (G, H) Only one unstimulated PBMC lacking T cell from both the twin healthy control (THC-P-T) and the unrelated healthy control (UHC-P-T) contained a “lipid droplet- like vesicle”. (TIF) [file pone.0272703.s015.tif]

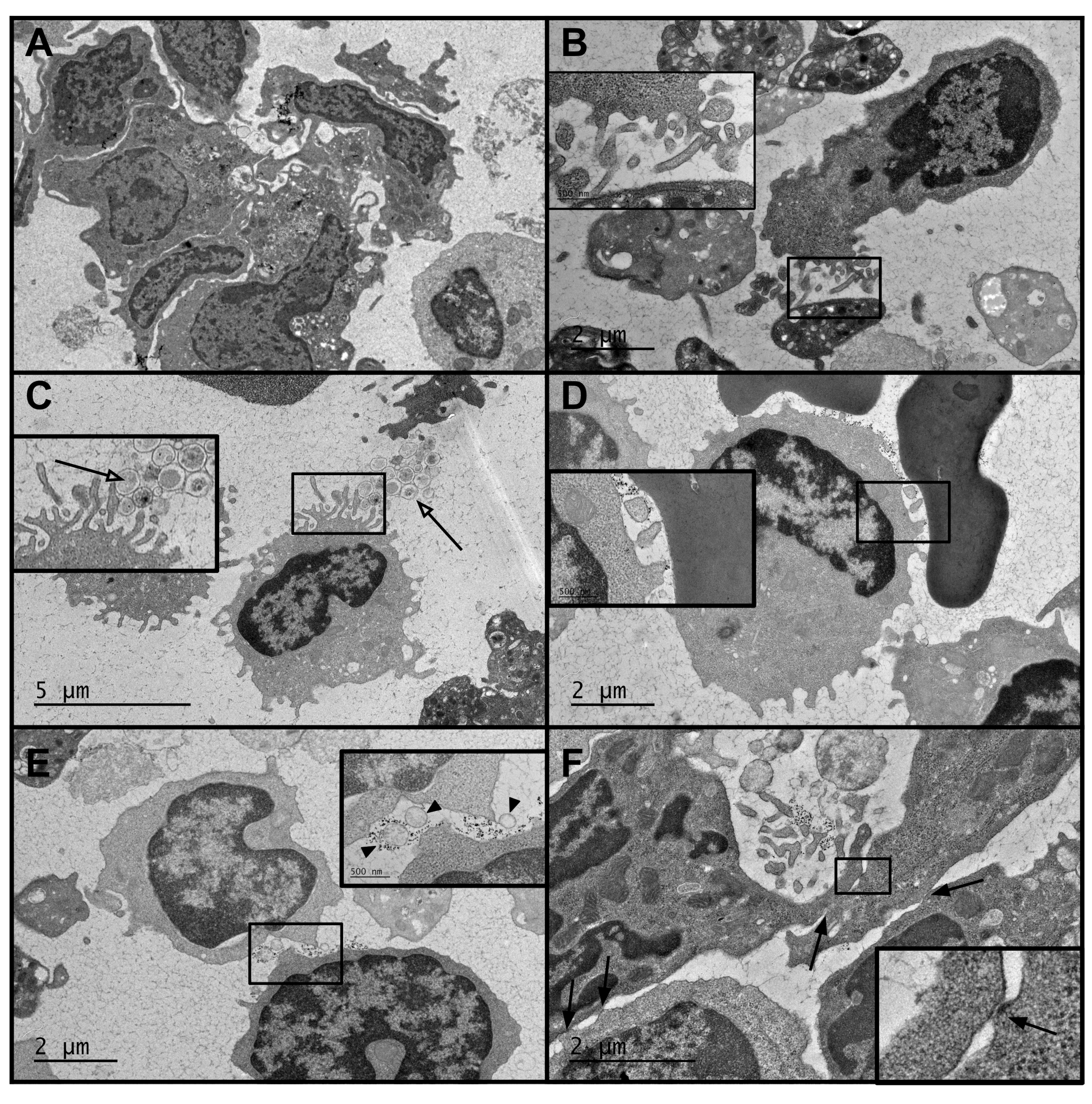

Supplement: S3 Fig — A) Interdigitating microvilli of stimulated T cells generating immune synapses. Microvilli can penetrate the glycocalyx, creating close-contact zones necessarily for immunological synapse function, and are thought to enable message transfer between cells, survey surfaces of antigen-presenting cells and carry T cell receptors. B) Displaying an immune cell along with several platelets, (inset) either representing PBMC microvilli formation near a platelet or platelet microparticles formation near a PBMC to form immune synapse. C) PBMC cell microvilli form budding vesicles constituting immunological synaptosomes, (inset) microvilli reaching out to potentially some platelet-derived microvesicles (PMVs) (open arrow). Microvesicles are important in cell–cell communication and cell differentiation. D) Exhibiting an immunological synapse between a PBMC and two red blood cells. Small particles can be seen in immune synapse junction, (inset) microvilli forming from the immune cell surface. E) The immune synapse between two immune cells, (inset) immune synapse is a site of intense vesicular trafficking, which can be seen as small electron dense particles around microvesicle-like structures (arrowhead) in immune synapse junction. F) Immune synapses (arrow) present between four immune cells. Microvesicles (open arrowhead) and small electron dense particles (arrowhead) can also be seen. (TIF) [file pone.0272703.s016.tif]

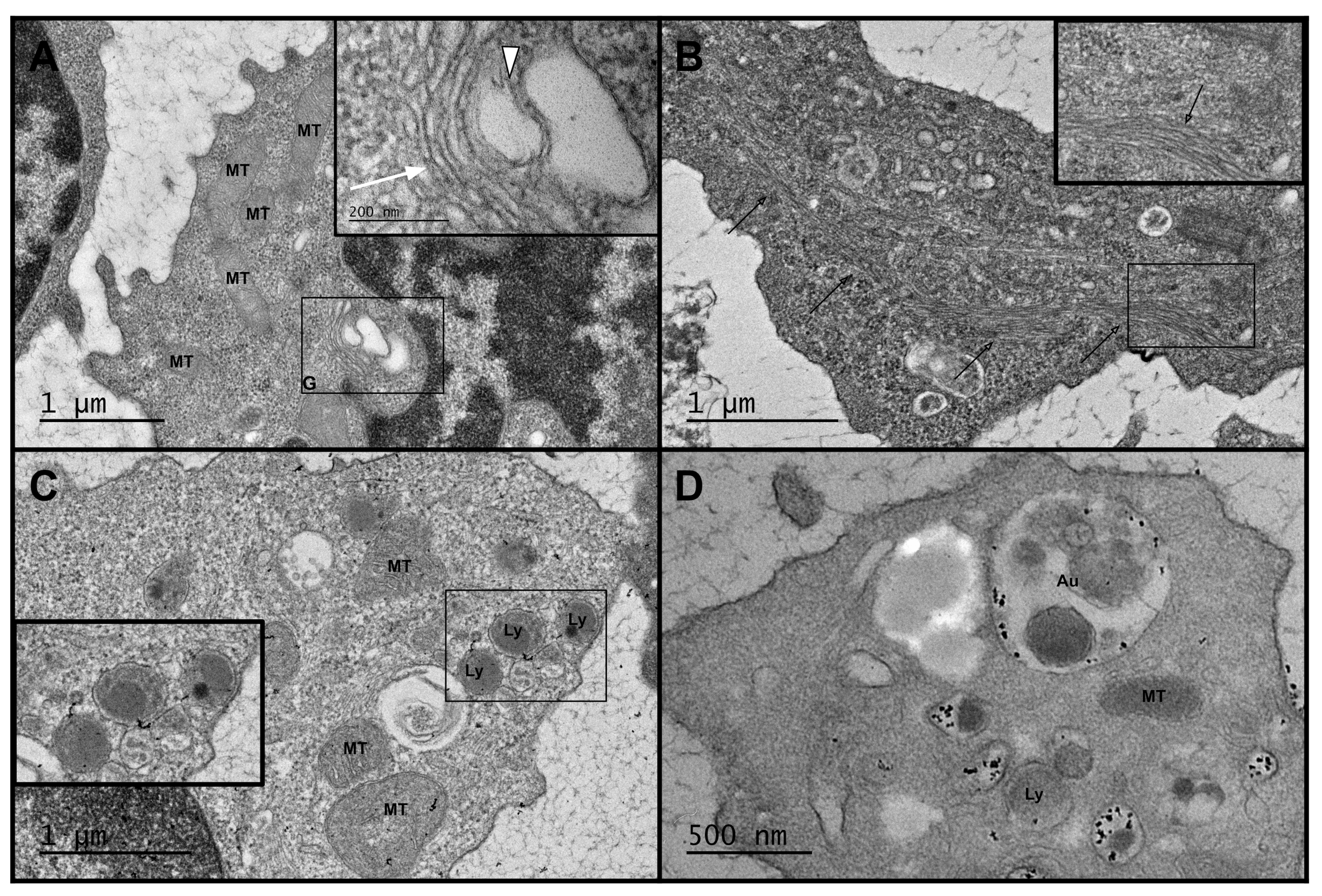

Supplement: S4 Fig — A) Golgi apparatus (G) near the nucleus and a few mitochondria (MT) can be seen, (Inset) ultrastructure of Golgi apparatus showing cisternae (white arrow) and large vesicles (white arrowhead). B) Endoplasmic reticulum (open arrow), (insert) ultrastructure of endoplasmic reticulum. C) Displaying a cluster of lysosome like-vesicles (Ly), (inset) typical ultrastructure of a lysosome showing spherical membrane bound organelles with an electron-dense cores indicative of high protein concentration. D) Autophagosome like-vesicle (Au) containing cytoplasmic materials. (TIF) [file pone.0272703.s017.tif]

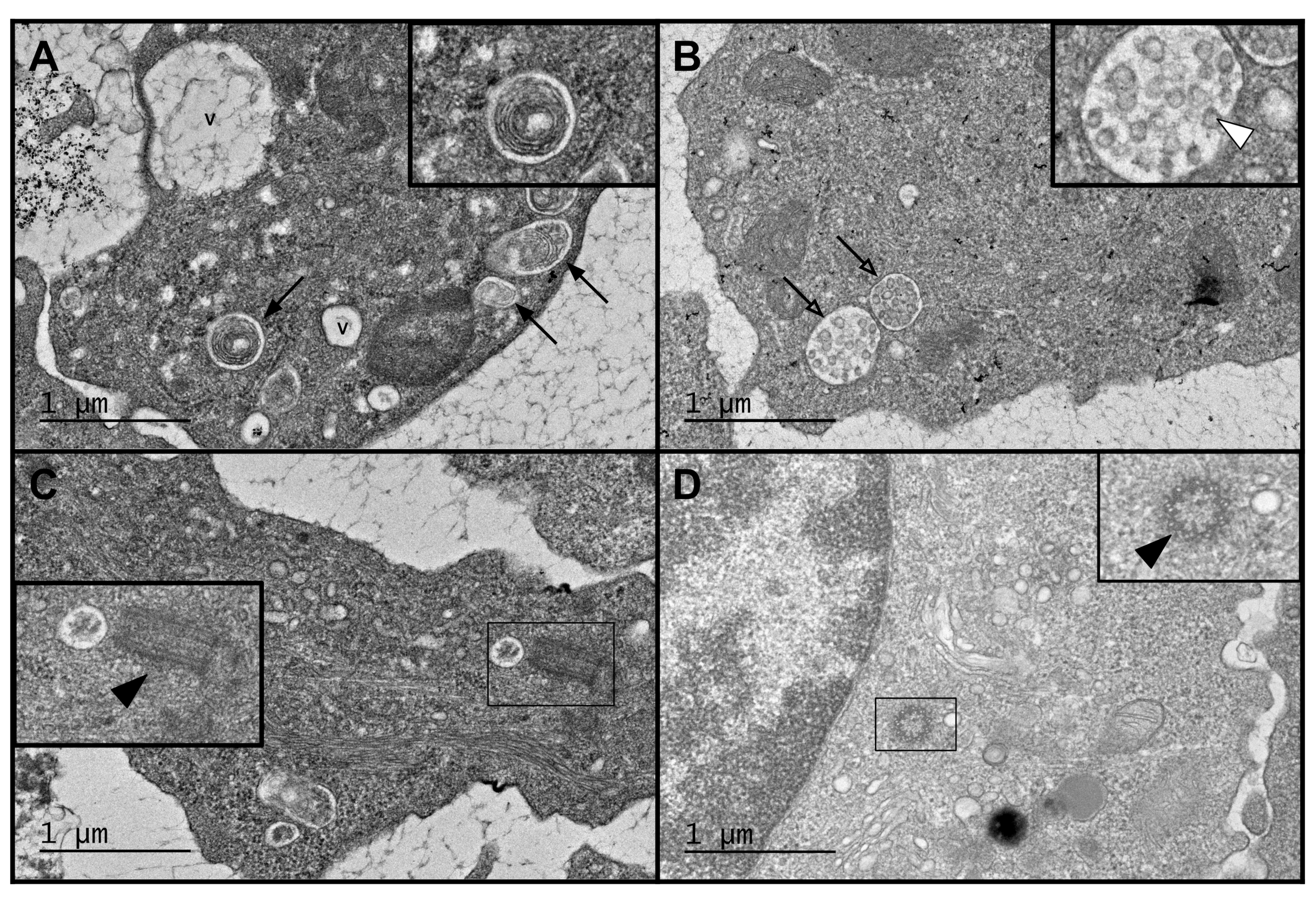

Supplement: S5 Fig — A) Vesicles (V) and multilamellar bodies (MLB) (arrows), which are membrane bound lysosomal vacuoles, (inset) ultrastructure of a MLB showing a membrane bound organelle containing concentric membrane layers. B) Multivesicular bodies (MVB) (open arrows), a particular type of endosome that contains membrane-bound intraluminal vesicles, (inset) ultrastructure of MVB with luminal vesicles (open arrowhead). C) Centriole (arrowhead) in a longitudinal orientation. D) Centriole cross section showing distinct microtubule triplet organization. (TIF) [file pone.0272703.s018.tif]
